# Role of opioid receptors in modulation of P2X receptor-mediated cardiac sympathoexcitatory reflex response

**DOI:** 10.1038/s41598-019-53754-6

**Published:** 2019-11-20

**Authors:** Liang-Wu Fu, Stephanie C. Tjen-A-Looi, Sherwin Barvarz, Zhi-Ling Guo, Shaista Malik

**Affiliations:** Susan Samueli Integrative Health Institute and Department of Medicine, School of Medicine, University of California at Irvine, Irvine, CA 92697 USA

**Keywords:** Autonomic nervous system, Acute coronary syndromes

## Abstract

Myocardial ischemia evokes powerful reflex responses through activation of vagal and sympathetic afferents in the heart through the release of ischemic metabolites. We have demonstrated that extracellular ATP stimulates cardiac sympathetic afferents through P2 receptor-mediated mechanism, and that opioid peptides suppress these afferents’ activity. However, the roles of both P2 receptor and endogenous opioids in cardiac sympathoexcitatory reflex (CSR) responses remain unclear. We therefore hypothesized that activation of cardiac P2 receptor evokes CSR responses by stimulating cardiac sympathetic afferents and these CSR responses are modulated by endogenous opioids. We observed that intrapericardial injection of α,β-methylene ATP (α,β-meATP, P2X receptor agonist), but not ADP (P2Y receptor agonist), caused a graded increase in mean arterial pressure in rats with sinoaortic denervation and vagotomy. This effect of α,β-meATP was abolished by blockade of cardiac neural transmission with intrapericardial procaine treatment and eliminated by intrapericardial A-317491, a selective P2X_2/3_ and P2X_3_ receptor antagonist. Intrapericardial α,β-meATP also evoked CSR response in vagus-intact rats. Furthermore, the P2X receptor-mediated CSR responses were enhanced by intrapericardial naloxone, a specific opioid receptor antagonist. These data suggest that stimulation of cardiac P2X_2/3_ and P2X_3_, but not P2Y receptors, powerfully evokes CSR responses through activation of cardiac spinal afferents, and that endogenous opioids suppress the P2X receptor-mediated CSR responses.

## Introduction

Metabolites released by the myocardium in the setting of myocardial ischemia activate cardiac vagal and sympathetic afferent endings in the ischemic myocardium. Activation of vagal sensory nerves induces vasodepressor responses and bradycardia^1,2^, while stimulation of sympathetic (spinal) afferent fibers results in cardiac sympathoexcitatory reflex (CSR) responses including increases in sympathetic outflow and arterial pressure, and tachyarrhythmias^3–6^. Increased arterial pressure can exacerbate ischemic events, which ultimately contribute to mortality and morbidity of patients with ischemic heart disease (IHD)^3,6,7^. Investigators have observed that ATP concentration is increased in the coronary effluent of hearts of experimental animals during myocardial ischemia^8,9^. Administration of ATP evokes a cardiac vagal depressor reflex by stimulating P2X_2/3_ purinergic receptor (P2X_2/3_R) located on ventricular vagal afferents^2,10,11^. However, there is no direct evidence available to show if activation of cardiac P2 receptor by extracellular ATP or its analogue is capable of evoking reflex cardiovascular responses. We have shown that blockade of cardiac P2 receptors attenuates the ischemia-induced increase in activity of cardiac spinal afferents^12^, suggesting that extracellular ATP may elicit vasopressor and tachycardia responses that contribute to the myocardial ischemia-mediated CSR responses.

Cardiac sympathetic sensory nerves include thinly myelinated Aδ- and unmyelinated C-fiber afferents and their cell bodies in the C7-T6 dorsal root ganglia (DRG)^13,14^. We and others have demonstrated that myocardial ischemia and many ischemic metabolites including bradykinin, thromboxane A2, reactive oxygen species, and among others can stimulate/sensitize these cardiac sensory nerves leading to CSR responses^3,15–17^. Extracellular ATP is one of the myocardial ischemic metabolites^8,9^ and exerts its actions through activation of purinergic 2 (P2) receptors, including P2X and P2Y^18^. P2X receptors are identified on the DRG in rats^18,19^. Investigators have shown that ATP and its analog α,β-methylene ATP (α,β-meATP) can elicit a reflex pressor response by stimulation of P2X receptors on group III and IV muscle afferents in cats^20,21^. Blockade of P2 receptors with pyridoxal phosphate-6-azophenyl-2′,4′-disulfonic acid (PPADS) eliminates the responses of muscle sensory nerve activity and the associated reflex pressor to intra-arterial injection of ATP analogs^21,22^. We have demonstrated that administration of P2X receptor agonist α,β-meATP increases activity of cardiac thinly myelinated and unmyelinated sympathetic afferents^12^. Additionally, P2Y receptors, including P2Y_1_, P2Y_2_, P2Y_4_, and P2Y_6_ subtypes, are expressed in sensory DRG neurons^23,24^. Application of P2Y1 receptor agonist ADP stimulates cutaneous sensory nerves, leading to pain sensation in human subjects^25–27^, which is consistent with our earlier observation that epicardial application of ADP stimulates cardiac spinal afferents^12^. Thus, we speculate that activation of cardiac P2 receptor including P2X and P2Y subtypes likely evokes CSR responses.

At supraspinal and spinal cord sites endogenous opioids generally function as atypical inhibitory neurotransmitters or neuromodulators that have been extensively studied^28–31^. However, the influence of the opiod on the peripheral nerve activity and the associated reflex responses has been investigated less extensively. In this respect, investigators have observed that opioids modulate peripheral sensory nerve activity through activation of opioid receptors on primary afferent fibers^32^. More recently, studies have shown that opioid receptors including µ-, δ-, and κ-subtypes are involved in modulation of peripheral somatic and visceral afferent activity^33–36^. Using an *in vitro* glabrous skin-nerve preparation, investigators observed that morphine reduces the excitatory responses of most C- and Aδ-fiber nociceptors to noxious mechanical and thermal stimuli in inflamed skin^33^. In addition, Tsuchimochi *et al*.^37^ documents that local administration of µ-receptor agonist significantly attenuates the exercise pressor reflex in the ischemic hindlimb, but the agonist exerts a minimal effect on the pressor reflex in the non-ischemic hindlimb. Our earlier studies have shown that peripheral opioid peptides inhibit the responses of cardiac sympathetic afferents to myocardial ischemia and ischemic metabolites^38^. However, it remains unclear if endogenous produced opioids modulate the activation of P2 receptor-mediated CSR responses.

The aim of the present study, therefore, was to investigate the role of P2 receptors, including P2X and P2Y subtypes, in cardiac sympathoexcitatory reflex responses and the influence of endogenous opioids on these CSR responses. We hypothesized that stimulation of P2 receptors evokes CSR responses through activation of cardiac spinal afferent mechanism, while the P2 receptor-mediated CSR responses are modulated by endogenous opioid peptides. Neurophysiological and pharmacological approaches were employed to test our hypotheses.

## Results

### Dose responses

**Figure 1:**
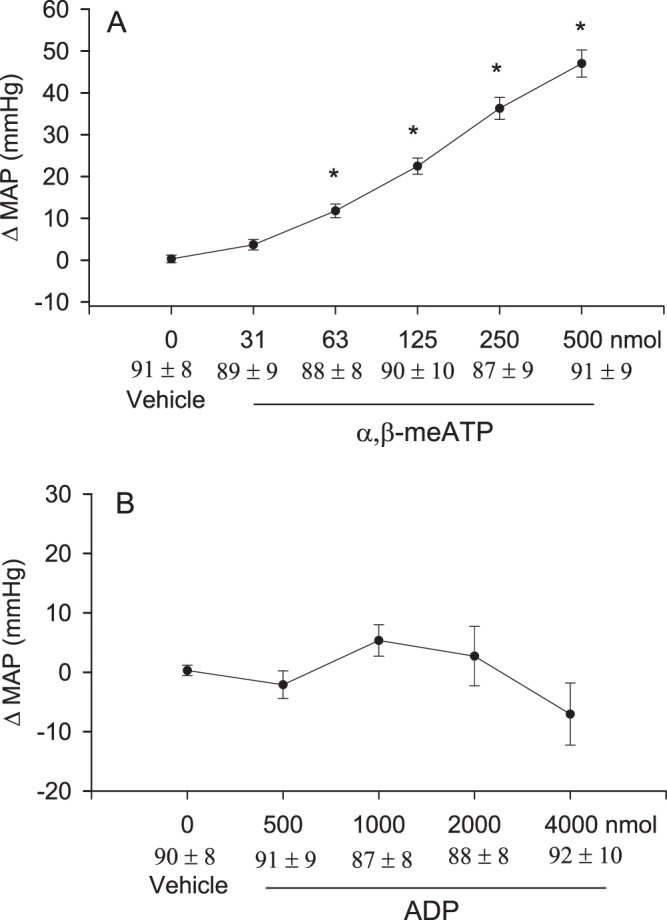
Line graph showing mean arterial pressure (MAP) responses to intrapericardial application of vehicle (PBS) and graded doses of α,β-meATP (n = 9, Panel A) and ADP (n = 9, Panel B) in barodenervated and vagotomized rats. Baseline MAP are shown below each dose. Values are means + SEM. *P < 0.05 compared with vehicle application.

Intrapericardial application of increasing doses of α,β-meATP, a selective P2X receptor agonist every 20 min evoked graded excitatory cardiovascular responses (Fig. 1A and supplemental Fig. 1A), while HR was unchanged in this group of barodenervated and vagotomized rats (n = 9). In contrast, intrapericardial application of ADP, a selective P2Y receptor agonist, did not alter mean arterial pressure (MAP) (Fig. 1B and supplemental Fig. 1B, P > 0.05) and heart rate (HR) in separate baro-vagal denervated rats (n = 9). Application of the vehicle (PBS) also did not alter MAP and HR. The baseline of MAP and HR before application of α,β-meATP were 91 ± 8 mmHg and 384 ± 14 beats/min.

### CSR responses to activation of P2X receptors before and after procaine

**Figure 2:**
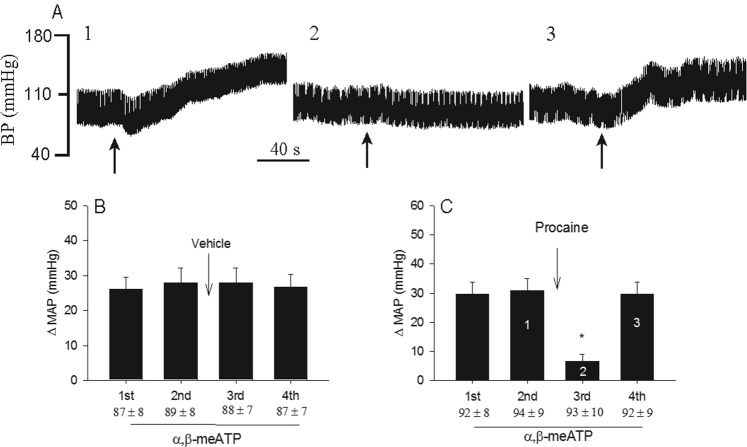
Arterial blood pressure responses to repeated intrapericardial α,β-meATP (125 nmol), before and after intrapericardial application of 2% procaine (80 µl, Panel B) in eight rats with barodenervation and vagotomy. Panel A: Original records of blood pressure responses in a rat that received intrapericardial α,β-meATP. 1 through 3 showing each BP tracing represent bars in panel C. (**B**) MAP responses to α,β-meATP before and after vehicle (PBS, n = 7). (**C**) MAP responses to α,β-meATP before and after procaine (n = 8). Baseline MAP are shown below each bar as means ± SEM. Columns and error bars represent means ± SEM. *P < 0.05 post-procaine vs. pre-procaine.

Representative tracings of blood pressure in top panels 1–3 in Fig. 2A display the changes of arterial pressure in response to intrapericardial α,β-meATP (125 nmol) before and after administration of procaine into pericardium of a baro-vagal denervated rat. Administration of α,β-meATP increased arterial blood pressure with MAP elevation by 27 mmHg (Fig. 2A1), which was eliminated by intrapericardial procaine (Fig. 2A2). The MAP response to application of α,β-meATP recovered to the pre-procaine level 40 min after third response (Fig. 2A3,C). The MAP responses to repeated intrapericardial applications of α,β-meATP (125 nmol) were consistent before and after intrapericardial application of vehicle in seven baro-vagal denervated rats (Fig. 2B and supplemental Fig. 2A). Application of α,β-meATP slightly decreased HR from 386 ± 18 to 382 ± 16 bpm (P > 0.05) in these rats. In contrast, blockade of cardiac neuronal transmission with intrapericardial procaine eliminated almost the MAP responses to application ofα,β-meATP (Fig. 2C and supplemental Fig. 2B) in eight other rats. The baselines of HR and MAP prior to each response were in a similar range (Table 1). Application of neither vehicle nor procaine itself changed MAP and HR (Table 1).

**Table 1:** Basal MAP and HR before and after application of inhibitor in baro-vagal denervated rats.

	n	MAP (mmHg)		HR (bpm)	
		Before	After	Before	After
Vehicle	7	90 ± 7	93 ± 8	387 ± 16	392 ± 15
Procaine	8	93 ± 8	90 ± 9	391 ± 18	383 ± 17
A-317491	7	95 ± 8	91 ± 8	389 ± 15	396 ± 18
Naloxone	8	91 ± 8	93 ± 7	394 ± 17	385 ± 16

Values are means ± SEM. MAP, mean arterial pressure; HR, heart rate.

### Effect of A-317491 on CSR responses to activation of P2X receptors

**Figure 3:**
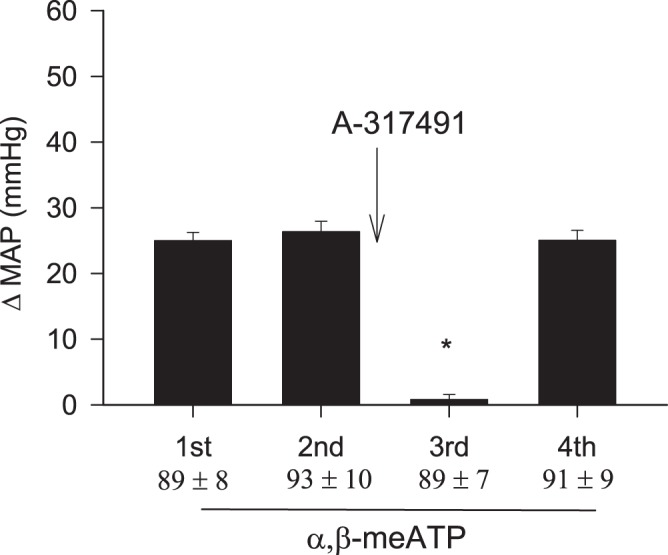
MAP responses to repeated intrapericardial α,β-meATP, before and after blockade of P2X_2/3_ and P2X_3_ receptors with A-317491 in seven barodenervated and vagotomized rats. The α,β-meATP (125 nmol) evoked MAP response was eliminated after intrapericardial application of A-317491 (800 nmol). Baseline MAP are shown below each bar as means ± SEM. Columns and error bars represent means ± SEM. *P < 0.05 post- A-317491 vs. pre- A-317491.

The increase in MAP induced by α,β-meATP (125 nmol) was eliminated reversibly by intrapericardial application of A-317491, a selective P2X_2/3_ and P2X_3_ receptor antagonist, in seven baro-vagal denervated animals (Fig. 3 and supplemental Fig. 3). Neither baseline MAP nor HR was altered by application of the P2X antagonist (Table 1). Cardiac α,β-meATP stimulation slightly increased HR by 15 ± 7 beats/min from a baseline of 388 ± 14 beats/min (P > 0.05), a response that was unaffected by A-317491 (13 ± 6 vs. 15 ± 7 beats/min, after vs. before A-317491, P > 0.05).

### Influence of naloxone on CSR responses to α,β-meATP

**Figure 4:**
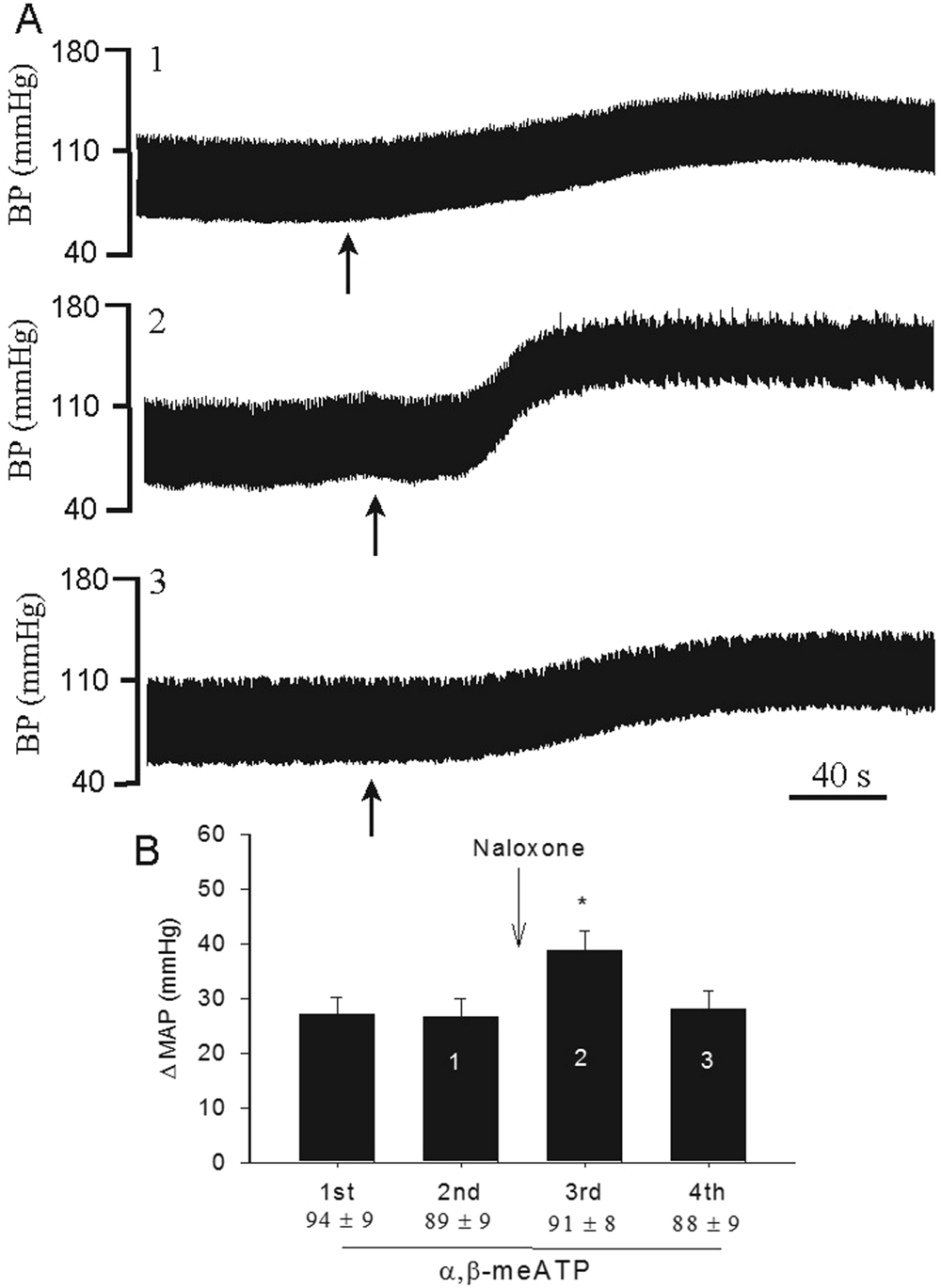
Bar graph displays cardiovascular responses to repeat intrapericardial α,β-meATP (125 nmol) before and after intrapericardial application of naloxone in 8 barodenervated and vagotomized rats. Numbers shown in bars (Panel B) correspond with BP response tracings shown in Panel A. Baseline MAP are shown below each bar as means ± SEM. Columns and error bars represent means ± SEM. *P < 0.05 post-naloxone vs. pre-naloxone.

Original records of arterial pressure responses to the intrapericardial α,β-meATP before and after blockade of opioid receptors with naloxone in an animal with barodenervation and vagotomy are shown in Fig. 4A. Administration of α,β-meATP increased MAP by 24 mmHg (Fig. 4A1), an effect that was enhanced by 58% (38 mmHg increase) after intrapericardial naloxone (Fig. 4A2). The α,β-meATP-induced MAP response was recovered 40 min after washout of intrapericardial naloxone (Fig. 4A3). In a group of baro-vagal denervated rats (n = 8), the α,β-meATP-evoked MAP response was significantly enhanced after intrapericardial naloxone (Fig. 4B and supplemental Fig. 4). The MAP response to intrapericardial α,β-meATP recovered after washout of naloxone (Fig. 4B). Administration of opioid receptor antagonist naloxone did not alter either basal MAP or HR (Table 1). Intrapericardial α,β-meATP stimulation slightly increased HR by 13 ± 6 beats/min from baseline of 387 ± 15 beats/min in this group, a response that was unaltered by naloxone application into pericardium (13 ± 6 vs. 16 ± 7 beats/min, after vs. before naloxone, P > 0.05).

### CSR responses to α,β-meATP before and after naloxone in animals without vagotomy

**Figure 5:**
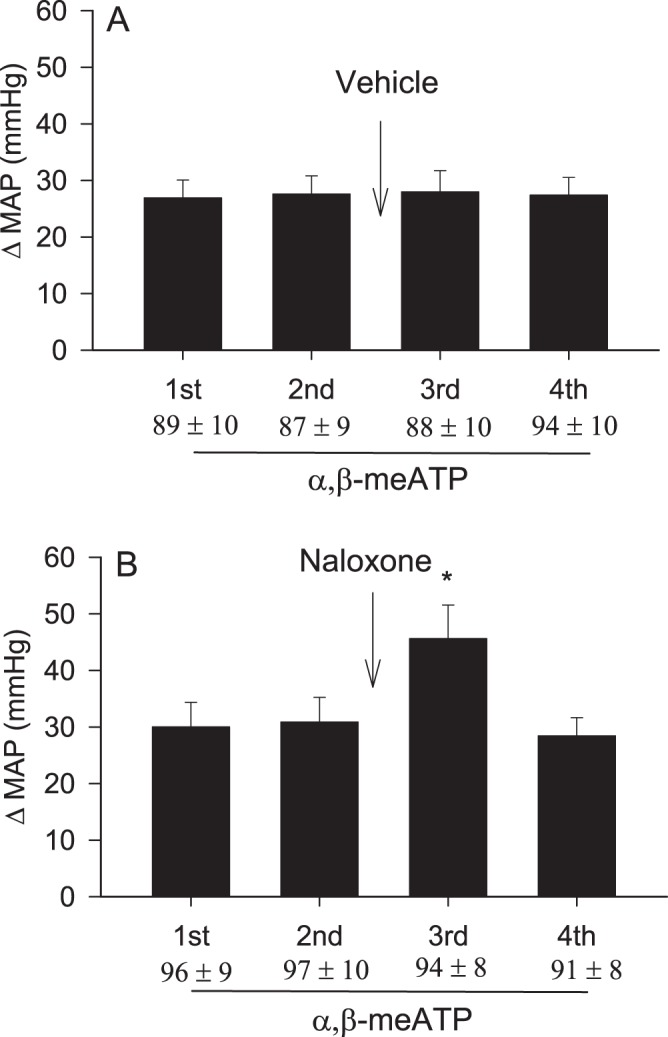
Arterial pressure responses to repeat intrapericardial α,β-meATP (125 nmol) before and after intrapericardial application of vehicle (PBS, n = 7, Panel A) and naloxone (n = 8, Panel B), a specific opioid receptor antagonist in vagus-intact rats. Baseline MAP are shown below each bar as means ± SEM. Columns and error bars represent means ± SEM. *P < 0.05 post-naloxone vs. pre-naloxone.

Baseline MAP (89 ± 5 mmHg) and HR (381 ± 11 beats/min) were similar in both group of rats, one with intact vagal and sympathetic afferent nerves and the other with baro-vagal denervation (Table 1). In the vehicle treated group (n = 7) repeat intrapericardial application of α,β-meATP evoked consistent increases in MAP (Fig. 5A and supplemental Fig. 5A). The magnitude of MAP response to α,β-meATP was similar between vagus-intact and vagotomized rats. In the opioid receptor antagonist treated group (n = 8), intrapericardial naloxone enhanced the α,β-meATP-evoked MAP response, which recovered 40 min after washout of naloxone (Fig. 5B and supplemental Fig. 5B). Intrapericardial α,β-meATP stimulation also increased but slightly HR by 13 ± 6 beats/min from baseline of 378 ± 12 beats/min in this group, a response that was unaltered by naloxone application into pericardium (13 ± 6 vs. 16 ± 7 beats/min, after vs. before naloxone, P > 0.05). Intrapericardial vehicle and naloxone did not alter the baseline of HR and MAP.

## Discussion

To our knowledge, this study is the first to assess whether stimulation of P2 receptors in the heart evokes excitatory cardiovascular reflex responses and if these reflex responses are modulated by peripheral opioids. It is known that the cardiac sympathoexcitatory reflex responses can deteriorate ischemic events leading to increase in morbidity and mortality in patients with ischemic heart disease^3,4,39,40^. In the present study, we observed that activation of P2X receptors with intrapericardial application of α,β-meATP, a mimetic of ATP and selective P2X receptor agonist provoked CSR reflex responses in a dose-dependent manner, while activation of P2Y receptor with ADP didn’t alter the CSR responses. The excitatory responses were eliminated after blockade of P2X receptors with their selective antagonist A-317491. Local blockade of cardiac afferent neurotransmission with intrapericardial application of the local anesthetic procaine also abolished the α,β-meATP induced hypertensive responses. In addition, blockade of opioid receptors with intrapericardial naloxone enhanced the P2X receptor-mediated reflexes in both vagus-intact and vagotomized animals. Taken together, these data indicate that activation of cardiac P2X receptors, but not P2Y, is capable of provoking cardiac sympathoexcitatory reflex responses through stimulation of cardiac sympathetic afferents. Endogenously produced opioids suppress the P2X-mediated CSR response through activation of peripheral opioid receptors.

The heart receives sympathetic and vagal efferent and afferent innervation, as well as intrinsic cardiac nerve supply. It is well known that stimulation of cardiac vagal afferents with ischemia, chemical, or electrical stimuli leads to vasodepressor and bradycardia responses, while activation of cardiac sympathetic afferents evokes vasopressor and tachycardia responses^3,5,13,41^. Myocardial ischemia increases extracellular ATP concentration^8,9^ and P2 receptors are located on parasympathetic sensory neurons like nodose ganglia and vagal afferent endings localized in lung and myocardium^11,42–44^. Studies reported that administration of ATP into coronary artery induces a cardiac vagal depressor reflex^2,11^. Hence, it is accepted that the increased ATP during myocardial ischemia mainly triggers cardiac vagal vasodepressor reflex by stimulating P2X_2/3_ receptors located on vagal sensory nerve terminals in the heart^2,10,11^. However, evidence also suggests that P2 receptors are located on spinal sensory neurons in the dorsal root ganglia and potentially on afferent terminals in the heart^8,9,42,45,46^, but the importance of extracellular ATP in provoking cardiac vasopressor and tachycardia responses remains unknown. The present study for the first time has provided evidence to demonstrate that intrapericardial application of α,β-meATP, an ATP analog, evokes pressor reflex responses in both vagotomized and vagus-intact rats (Figs 2 and 5). The α,β-meATP-mediated vasopressor reflex can be eliminated by blockade of cardiac afferent neurotransmission with intrapericardial application of local anesthetic drug procaine, suggesting that intrapericardial application of α,β-meATP highly likely stimulates cardiac sympathetic sensory nerve endings that is located more superficial and nearer to the epicardial surface of the heart^47,48^. This is consistent with our previous observation that epicardial application of ATP and α,β-meATP excites ischemically sensitive cardiac sympathetic afferents^12^.

Action of extracellular ATP is mediated by the P2 receptors including ionotropic P2X and metabotropic P2Y families^18^. P2X receptor activation causes ion flux through the ligand-gated ion channels in cell membrane, while activation of the P2Y receptor essentially causes an intracellular-reaction cascade through a G-protein coupled mechanism. In the nervous system, both the P2X and P2Y receptor subtypes are presented in the DRG^23,24,45,46^ and these DRG P2 receptors could be transported to the axonal nerve ending in the heart similar to the transport of DRG vanilloid receptors^49^. In the present study, we have observed that excitation of P2Y receptor with ADP fails to elicit vasopressor response although our earlier studies have shown that ADP excites cardiac sympathetic afferent^12^. The following factors are potentially responsible for this discrepancy. First, multiple P2Y receptor subtypes including P2Y1,2,4,6 are expressed in rat heart and coronary arteries and the P2Y receptor mediates inhibition of the heart^10,50^. ADP can induce relaxation of coronary small arteries through activation of P2Y receptors^51,52^. Second, rapid breakdown of ADP to adenosine, which in turn leads to direct negative inotropic and chronotropic effect through action on P1 purinoceptors located in the heart^53^. Last and most importantly, the cardiac sympathetic afferent response to ADP occurs at the sensory nerve fiber site. However, to evoke cardiac-sympathoexcitatory reflex responses it is necessary to excite the entire CSR reflex arc neural pathway that includes sensory neuron and fiber, integrative center(s) in the brain and spinal cord, sympathetic efferent nerve and effector organ in the cardiovascular system. It is possible that the ADP-induced afferent activation is insufficient to activate the entire neural pathway involved in the CSR reflex arc.

In contrast, the present study has documented that activation of P2X receptors with α,β-meATP evokes CSR responses, and this is consistent with our previous data that stimulation of P2X receptor activates cardiac sympathetic afferents^12^. Moreover, the α,β-meATP induced vasopressor response is eliminated by selective blockade of P2X_2/3_ and P2X_3_ receptors with A-317491. Previous studies have documented that among the members of the P2X receptor family, the heteromeric P2X_2/3_ as well as homomeric P2X_1_, P2X_2_ and P2X_3_ receptors are sensitive to α,β-meATP^54^. By using A-317491, a potent and selective P2X_2/3_ and P2X_3_ receptor antagonist^55^, our data suggest that the P2X_2/3_ and P2X_3_ receptors located on cardiac sympathetic sensory neurons, but not P2Y receptors, are involved in the ATP-evoked CSR responses.

In addition to the P2X receptor-mediated CSR responses, the P2X receptor-mediated direct action as well as the P2Y and P1 receptor-induced effects in the heart also may contribute to the interplay between purinergic and adrenergic signaling in regulation of heart. In this respect, studies indicate that mRNA and protein of all P2X subtypes are expressed on cardiac myocytes^56^. ATP can produce positive chronotropic and inotropic effects on the heart and induce contractile responses of coronary arteries through direct activation of P2X receptors^57^. On the other hand, multiple P2Y receptor subtypes including P2Y1,2,4,6 are expressed in rat heart and coronary arteries^10,50^. Activation of P2Y receptors directly inhibits heart and adenosine-P1 receptors can induce negative inotropic and chronotropic effect and anti-β adrenergic actions in the heart^53,58^. Studies also have shown that ATP and its rapid breakdown products such as ADP and adenosine evoke endothelium-dependent or independent vasodilation in isolated human coronary arteries and other arteries through activation of P2Y or P1 receptors^51,52^. Additionally, ATP and noradrenaline as co-transmitters are released by sympathetic efferent nerves^10,52^. The released ATP inhibits the release of noradrenaline in the heart through its action on P2Y receptors located on the sympathetic terminals^59,60^, while noradrenaline can suppress the release of ATP from sympathetic nerves^59^. Hence, a physiological interplay between purinergic and adrenergic signaling in the heart warrants further studies.

Previously, multiple studies have shown that opioid receptor including µ-, δ-, and κ-receptors are located on small-, medium- and large-diameter sensory neurons in the DRG, nodose and trigeminal ganglia of animals and humans^32,61,62^. Fields and his colleagues^32^ have shown multiple subtypes of opioid receptors located on primary afferent fiber terminals. The three subtypes of opioid receptors belong to the superfamily of G protein-coupled receptor (GPCR). Investigators have documented that opioids induce variable somatic and visceral sensory neural responses. In this regard, stimulation of opioid receptors on pelvic and gastric vagal sensory nerves suppresses visceral pain^63,64^. Others reported that opioid peptides excite rat mesenteric afferents and mouse DRG neurons, which can be eliminated by blockade of opioid receptors^65,66^. We found that endogenous opioids modulate the responses of cardiac sympathetic afferents to exogenous ATP and myocardial ischemia^38^, suggesting that endogenous opioids likely suppress the P2X receptor activation-evoked CSR responses by inhibiting the excitability of ischemically sensitive cardiac spinal afferents through stimulation of opioid receptors located on the cardiac spinal afferent terminals. Our speculation was consistent with the findings of other investigators. First, He and his colleagues^67^ have shown that µ-opioid receptors are expressed and co-localized with TRPV1 receptors on the cardiac sensory nerve terminals. Second, Chizhmakov and his colleagues^68^ reported that leu-enkephalin and morphine inhibit ATP-evoked excitation of somatic C-fiber sensory nerves through a GPCR-dependent mechanism, an effect that can be reversed by naloxone. Our speculation also is supported by our own findings showing that specific blockade of opioid receptors with naloxone enhanced the α,β-meATP-evoked vasopressor response in both vagus-intact and vagotomized rats. Moreover, stimulation of opioid receptors with intrapericardial application of DAMGO (400 nmol), an opioid receptor agonist, reduced the P2X receptor-mediated CSR responses by 48% in three vagus-intact rats in our pilot study (unpublished data). Additionally it is interesting to note that in rats with both vagal and sympathetic cardiac nerves intact, intrapericardial naloxone similarly exaggerated the α,β-meATP evoked hypertensive responses, although opioid receptors also are located on vagal afferents and nodose ganglion neurons^63,64,68^. Together, the present data suggest that blockade of opioid receptors by intrapericardial naloxone mainly enhances the α,β-meATP induced excitatory cardiac-cardiovascular reflex responses in rats.

### Physiological and clinical implications

Clinical observations illustrate that angina pectoris can be accompanied by either vasopressor and tachycardia or vasodepressor and bradycardia^69–71^ since myocardial ischemia through release of many mediators strongly stimulates both vagal and sympathetic cardiac sensory nerve fibers^11–13,41,72^. Excitation of myocardial sympathetic afferents (i.e., cardiac spinal afferent) leads to the excitatory cardiovascular reflexes including hypertension and tachycardia^1,3,5,13^, while stimulation of cardiac vagal afferents causes vasodepressor and bradycardia^2,41^. Anatomic studies have shown that P2X receptors are located on both cardiac vagal and sympathetic sensory nerves^2,10–12,45,46^. Cardiac vagal afferent fibers are located nearer to the endocardial layer^48^, while sympathetic afferent fibers mainly are located on more superficial and nearer to the epicardial surface of the heart^47,48^. In addition, studies have shown that ATP breakdown occurs very rapidly and its half-life is about 0.2 s when perfused in the circulation^73,74^. Therefore, clinicians likely observe hypotension and bradycardia responses when patients suffer from subendocardial ischemia because of regional ischemia increasing ATP locally, which in turn stimulates cardiac vagal afferents. This is supported by previous studies conducted by Xu and his colleagues^2^. In contrast, when transmural ischemia occurs in patients, hypertension and tachycardia responses are observed as the locally increased ATP largely activates cardiac sympathetic afferents located closer to the epicardial surface of the heart, which is supported by both the present results and our earlier study^12^.

In summary, the novel evidence generated from the present study demonstrate that activation of P2X_2/3_ and P2X_3_ receptors, but not P2Y receptors, evokes cardiac hypertension response through stimulation of cardiac sympathetic sensory nerve fibers, the response that can be reduced by endogenous opioids through excitation of opioid receptor mechanisms. Since myocardial ischemia leads to release of both ATP and opioids into the extracellular space in the heart^8,9,75^, the interactions between opioids and ATP highly likely contribute to the net cardiovascular responses during myocardial ischemia. These new findings extend our knowledge of ischemic mediators like extracellular ATP produced during myocardial ischemia in stimulating cardiac sensory neurons-cardiovascular reflex responses, while endogenous opioids in suppressing the CSR responses through activation of opioid receptors located on cardiac spinal afferent terminals^67^, which may help cardiologist to develop an innovative therapy for reducing morbidity and mortality in patients with ischemic heart diseases. For instance, we have shown that electroacupuncture can reduce gastric distension-induced excitatory cardiovascular reflexes through activating endogenous opioid pathways^28,30^, suggesting a possibility that acupuncture could attenuate the CSR reflex response, which needs further exploration. The role of opioid receptor subtypes on the P2X receptor-mediated CSR responses also requires further exploration.

## Methods

### Surgical preparation

All experimental preparations and protocols were reviewed and approved by the Animal Care and Use Committee at the University of California, Irvine. The investigation conformed to the American Physiological Society’s “Guiding Principles in the Care and Use of Animals.” Adult Sprague-Dawley (SD) male rats (350–550 g) were anaesthetized initially with ketamine (100 mg/kg, im) followed by α-chloralose (50–60 mg/kg, iv). Additional doses of α-chloralose (25–30 mg/kg, iv) were given as necessary to maintain an adequate level of anesthesia assessed by observing the absence of a conjunctival reflex. A femoral vein was cannulated to administer drugs and fluids. Systemic arterial blood pressure was monitored by a pressure transducer attached to a carotid artery cannula. The trachea was intubated and respiration was maintained artificially (model 661, Harvard ventilator, Ealing, South Natick, MA, USA). Rats were ventilated with room air supplemented with 100% O_2_ through the respirator. Arterial blood gases and pH were measured with a blood gas analyzer (ABL 5, Radiometer America, Inc., West Lake, OH) and were maintained within physiological limits (PO_2_ > 100 mmHg, PCO_2_ 30–40 mmHg, pH 7.35–7.45) by adjusting the respiratory rate, tidal volume or by administering NaHCO_3_ (1 M, iv). Body temperature was monitored by a rectal thermistor and maintained at 36–38 °C with a circulating water-heating pad and heat lamp.

### Sinoaortic denervation and cervical vagotomy

To eliminate the influence of vagal cardiac afferents that could mask the CSR responses to stimulation of sympathetic afferents and minimize the BP “buffering” action of arterial baroreceptors, bilateral cervical vagotomy and sinoaortic denervation with sectioning of carotid sinus nerves were conducted in rats used in first four protocols (see in following protocols), as described previously^3,76^. Vagotomy was not performed in rats used in the last protocol (described in protocols section) for examining the CSR responses in vagus-intact animals. The barodenervation was verified by noting the absence of the normal decrease of heart rate (HR) in response to ~40-mmHg increase in arterial BP induced by administration of phenylephrine (10 µg/kg, iv)

### Intrapericardial catheter insertion

To administer chemicals to the heart, a catheter was placed in the pericardial sac as previously described^17,77^. In brief, a high midline thoracotomy (the collarbone and the first two ribs) was conducted to expose the thymus and heart. A polyethylene-50 (PE) tubing with 6–8 small holes in the distal end was inserted into the pericardial space over the left ventricle through a small incision made on the thymus gland (on the midline aspect of the thymus). The catheter was then sealed into the thymus and pericardium by suturing together the two thymus lobes and the surrounding muscle tissue with a silk suture to prevent any leaks from pericardium. Various chemical solutions that can stimulate or inhibit cardiac afferent nerve endings described in the following protocols were injected through the PE-50 catheter into the pericardial space. At end of experiment, we injected 80 µl of 2% Chicago Sky blue into the pericardial space in each rat, and leakage of dye from the pericardium was assessed visually at autopsy. Leakage occurred in ~4% of all rats and the animals with leakage from the pericardium were excluded from this study.

#### Drugs

In this study we used α,β-meATP (0.75–12.5 mM), a selective P2X receptor including P2X_1_, P2X_2/3_, and P2X_3_ subtypes agonist^12,20,54^; A-317491 (10 mM), a selective P2X_2/3_ and P2X_3_ receptor antagonist^55^; and ADP, a selective P2Y receptor agonist^26^. Naloxone (100 mM), specific opioids receptor antagonist^33,38^ and 2% procaine, local anesthetic drug^3,78^ also were used.

Each drug was dissolved in phosphate buffer solution (PBS, pH 7.35) to a stock concentration. The pH of working solution of each drug was adjusted with 1 M of NaHCO_3_ [8.4% (wt/vol)] to a final value of 7.35. Procaine, A-317491 and α,β-meATP were purchased from Sigma-Aldrich (St. Louis, MO). Naloxone was purchased from Tocris (Minneapolis, MN). The stock solution was prepared weekly and stored in a −20 °C freezer and the working solution was prepared daily.

### Experimental protocols

#### Dose responses of reflexes to α,β-meATP

In this protocol we examined CSR responses following activation of P2X receptors with intrapericardial application of α,β-meATP and activation of P2Y receptors with intrapericardial ADP. After completion of the surgical preparation including denervation, a minimum of 45 minutes was allowed for stabilization of arterial pressure. The CSR responses including arterial blood pressure and heart rate (HR) to application of α,β-meATP were recorded with injection of 40 µl of various doses of α,β-meATP or PBS (vehicle) into the pericardial space in nine barodenervated and vagotomized rats. The vehicle (i.e., PBS) and α,β-meATP were applied randomly. A dose-response curve was generated with five doses of α,β-meATP (31, 63, 125, 250, and 500 nmol). α,β-meATP is a selective P2X_1_, P2X_2/3_, and P2X_3_ receptor agonist and mimetic of ATP that is produced during myocardial ischemia and participates in activation of cardiac spinal afferents^12^. In separate group (n = 9), the CSR responses to randomly intrapericardial ADP with four doses (500, 1000, 2000, 4000 nmol) were recorded. ADP is a selective P2Y receptor agonist. The heart was washed with intrapericardial injection of 100 µl of warm saline (35 °C) three times to wash out the drug after each application. To prevent tachyphylaxis, recovery periods of at least 20 min were provided between consecutive stimuli.

#### α,β-meATP + procaine

The influence of blockade of cardiac nerve transmission with procaine on the CSR responses to cardiac P2X receptor activation in bilateral barodenervated and vagotomized animals was examined in this protocol. After stabilization, α,β-meATP (125 nmol, 40 µl) was injected into the pericardial space to evoke repetitive reflex increases in BP and HR. Warm saline (100 µl) was applied intrapericardially three times to wash out α,β-meATP after each application of the P2X receptor agonist. To prevent tachyphylaxis, recovery periods of at least 20 min were provided between consecutive stimuli. In this protocol, intrapericardial α,β-meATP was applied 4 times over a period of at least 100 min. After the first two consecutive application of α,β-meATP, 80 µl of 2% procaine were injected into the pericardial sack of eight rats. Next, third intrapericardial α,β-meATP was conducted 5 min after procaine, which was 20 min after the second dose of α,β-meATP. Previous studies have demonstrated that this dose of procaine eliminates cardiac reflex responses by blocking cardiac afferent neurotransmission^3,78^ since cardiac spinal afferent nerve endings are located mainly in the epicardial layers of the myocardium^48^. 40 minutes later, a fourth application of α,β-meATP was performed to observe a recovery of BP/HR responses to α,β-meATP. To evaluate the reproducibility of cardiovascular reflex responses to α,β-meATP, seven additional rats were studied used as time control group. Each animal in this group was treated identical with exception that an intrapericardial application of vehicle (PBS, 80 µl) was used in place of procaine. This time control group also served as control for the following two protocols.

#### α,β-meATP + A-317491

To evaluate the influence of blockade of P2X receptors on CSR responses to α,β-meATP, we recorded BP and HR responses following repeated application of α,β-meATP before and after intrapericardial application of A-317491, a selective P2X_2/3_ and P2X_3_ receptors antagonist in bilateral barodenervated and vagotomized animals (n = 7). After stabilization, in a similar fashion as above mentioned procaine protocol, 80 µl of A-317491 (800 nmol) was injected into pericardium after first two consecutive applications of α,β-meATP. The third intrapericardial α,β-meATP was conducted 5 min after A-317491, which was 20 min after the second application of α,β-meATP. Previous studies have demonstrated that A-317491 at this dose reduces α,β-meATP-induced pressor response by selective antagonism of P2X_2/3_ and P2X_3_ receptors^55^. Following each application of α,β-meATP, the heart was washed three times with 100 µl of warm saline. 40 minutes later, a fourth application of α,β-meATP was performed to observe a recovery of BP/HR responses to α,β-meATP.

#### α,β-meATP + naloxone

In eight animals, we examined the influence of blockade of opioid receptors with naloxone, a specific opioid receptor antagonist, on the cardiovascular responses (BP and HR) to stimulation of P2X receptors with α,β-meATP. Following each application of α,β-meATP, the heart was washed three times with 100 µl of warm saline. In a similar fashion as the A-317491 protocol, after the first two consecutive intrapericardial α,β-meATP, 80 µl of naloxone (8 µmol) was injected into pericardium and third intrapericardial α,β-meATP was performed 5 min after naloxone and 20 min after the second application of α,β-meATP. We have demonstrated that this dose of naloxone enhances cardiac afferent activity in response to ischemia by blockade of opioid receptors^38^. Next, α,β-meATP was reapplied into pericardium 40 minutes after the third application of α,β-meATP, to allow for a recovery of the reflex responses comparable to the control level.

#### α,β-meATP + naloxone in vagus-intact rats

To determine if intrapericardial α,β-meATP induce cardiac vagal reflex responses including vasodepressor and bradycardia that may affect the P2X receptor activation-mediated CSR responses and if naloxone modulate the α,β-meATP-evoked responses, we recorded BP and HR responses to repeated intrapericardial application of α,β-meATP in two groups of rats without bilateral vagotomy. In an identical manner as protocol 4, vehicle in first group (n = 7) and naloxone (8 µmol) in second group (n = 8) of rats were applied into pericardial space after the first two intrapericardial α,β-meATP.

### Data analysis

Arterial blood pressure and HR were recorded with a Spike 2 data-acquisition system (CED micro 1401 mkII) and stored on a computer hard drive (Dell). Mean arterial pressure (MAP) is expressed in mmHg and HR is expressed in beats per minute. Data are expressed as means ± SEM. The Shapiro-Wilk test was used to determine if the data were distributed normally. Normally distributed data in all protocols were compared with either a Student’s paired *t*-test for paired data or a one way repeated-measures ANOVA followed by the Holm-Sidak’s post hoc test. All statistical calculations were performed with SigmaStat software (Jandel scientific Software, San Rafael, CA). Values were considered to be significantly different when P < 0.05.

## Supplementary information

Supplementary information

## Data Availability

The data sets generated and analyzed during the current study are available from the corresponding author on reasonable request.
