# Supplementary material for: Role of opioid receptors in modulation of P2X receptor-mediated cardiac sympathoexcitatory reflex response
Source: Sci Rep. 2019 Nov 20;9:17224. doi: 10.1038/s41598-019-53754-6 (PMC6868205; doi:10.1038/s41598-019-53754-6)
Supplement: Supplementary file 1 — Supplementary information [file 41598_2019_53754_MOESM1_ESM.pdf]

Role of opioid receptors in modulation of P2X receptor-mediated cardiac sympathoexcitatory reflex response

Liang-Wu Fu*, Stephanie C. Tjen-A-Looi, Sherwin Barvarz, Zhi-Ling Guo, and Shaista Malik

Susan Samueli Integrative Health Institute and Department of Medicine, School of Medicine, University of California at Irvine, Irvine, CA, 92697, USA

Address for correspondence:

Liang-Wu Fu, M.D., Ph.D.

Susan-Samueli Institute for Integrative Health

C240 Medical Sciences I

University of California, Irvine

Irvine, California 92697

(949) 824-8161

Email : lwfu@uci.edu

Supplemental material

Supplemental Figure

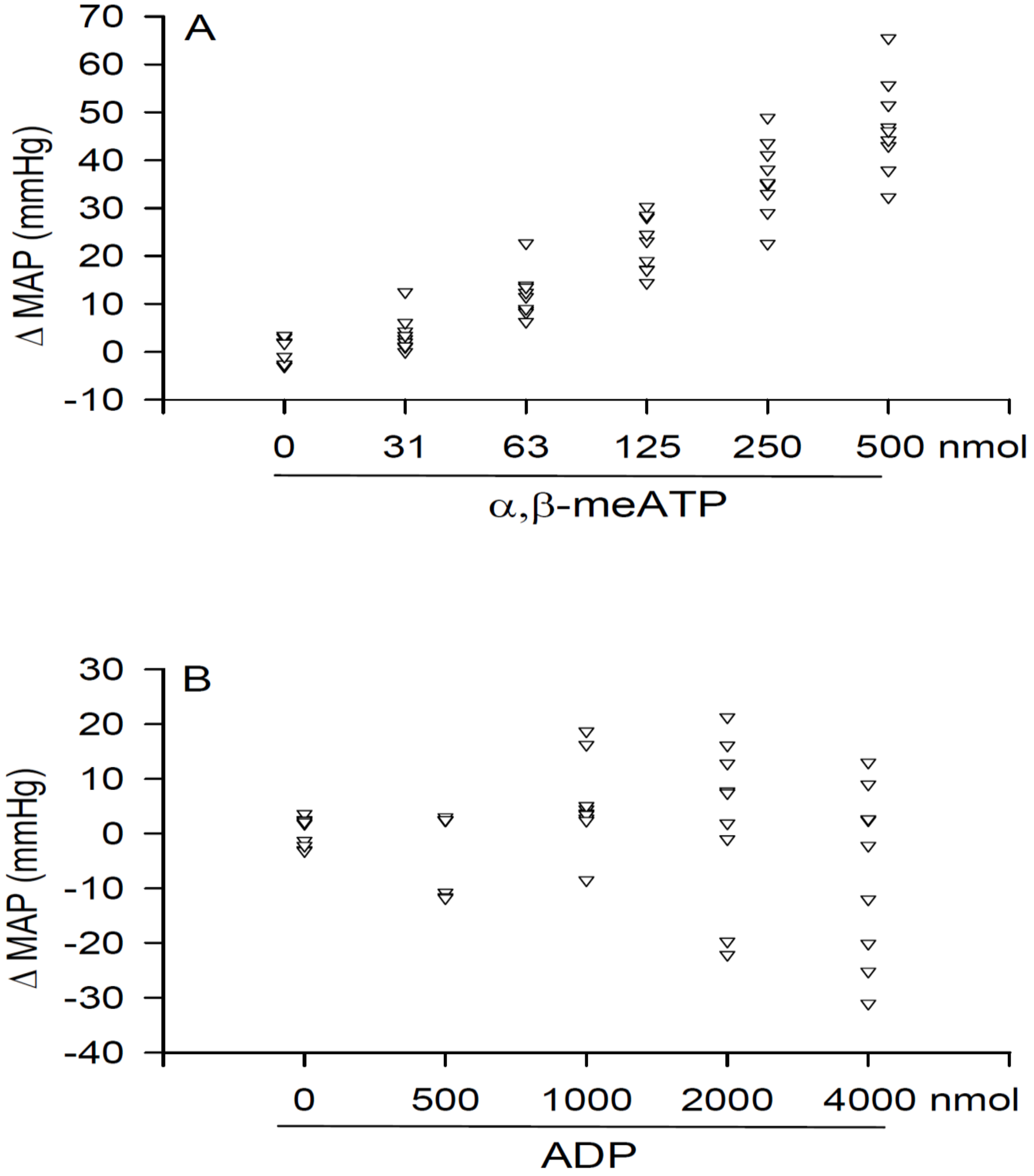

Fig. 1. Graphs represent individual data points showing mean arterial pressure (MAP) responses to intrapericardial application of vehicle (PBS) and graded doses of α,β-meATP (Panel A, n=9) and ADP (Panel B, n=9) in barodenervated and vagotomized rats.

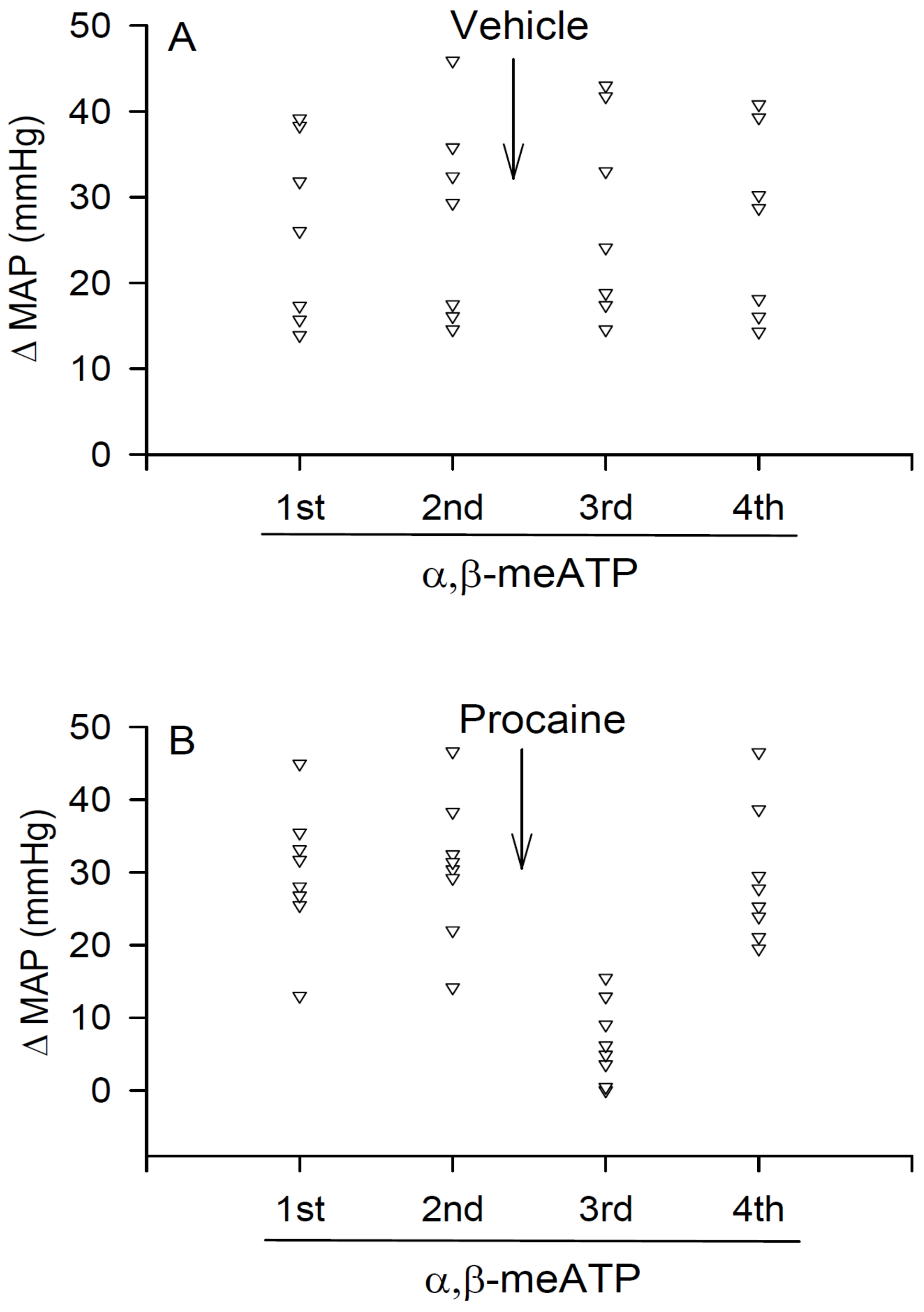

Fig. 2. Individual data point graphs show MAP responses to repeated intrapericardial α,β-meATP (125 nmol), before and after intrapericardial application of vehicle (PBS, Panel A) in seven rats and 2% procaine (80 µl, Panel B) in eight rats with barodenervation and vagotomy.

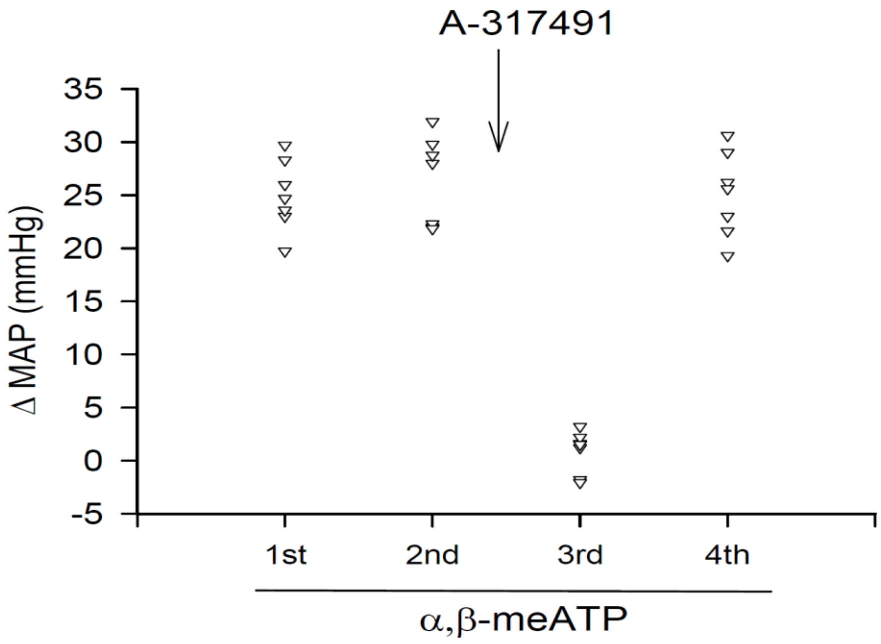

Fig. 3. Individual data point graphs display MAP responses to repeated intrapericardial α,β-meATP, before and after blockade of $P2X_{2/3}$ and $P2X_3$ receptors with A-317491 in seven barodenervated and vagotomized rats.

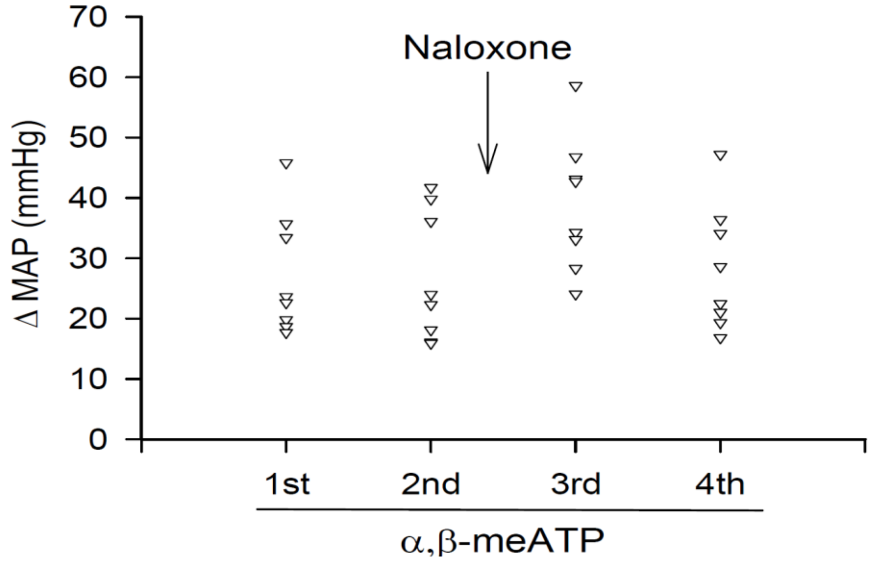

Fig. 4. Graphs represent individual data points showing MAP responses to repeat intrapericardial α,β-meATP (125 nmol) before and after intrapericardial application of naloxone in 8 barodenervated and vagotomized rats.

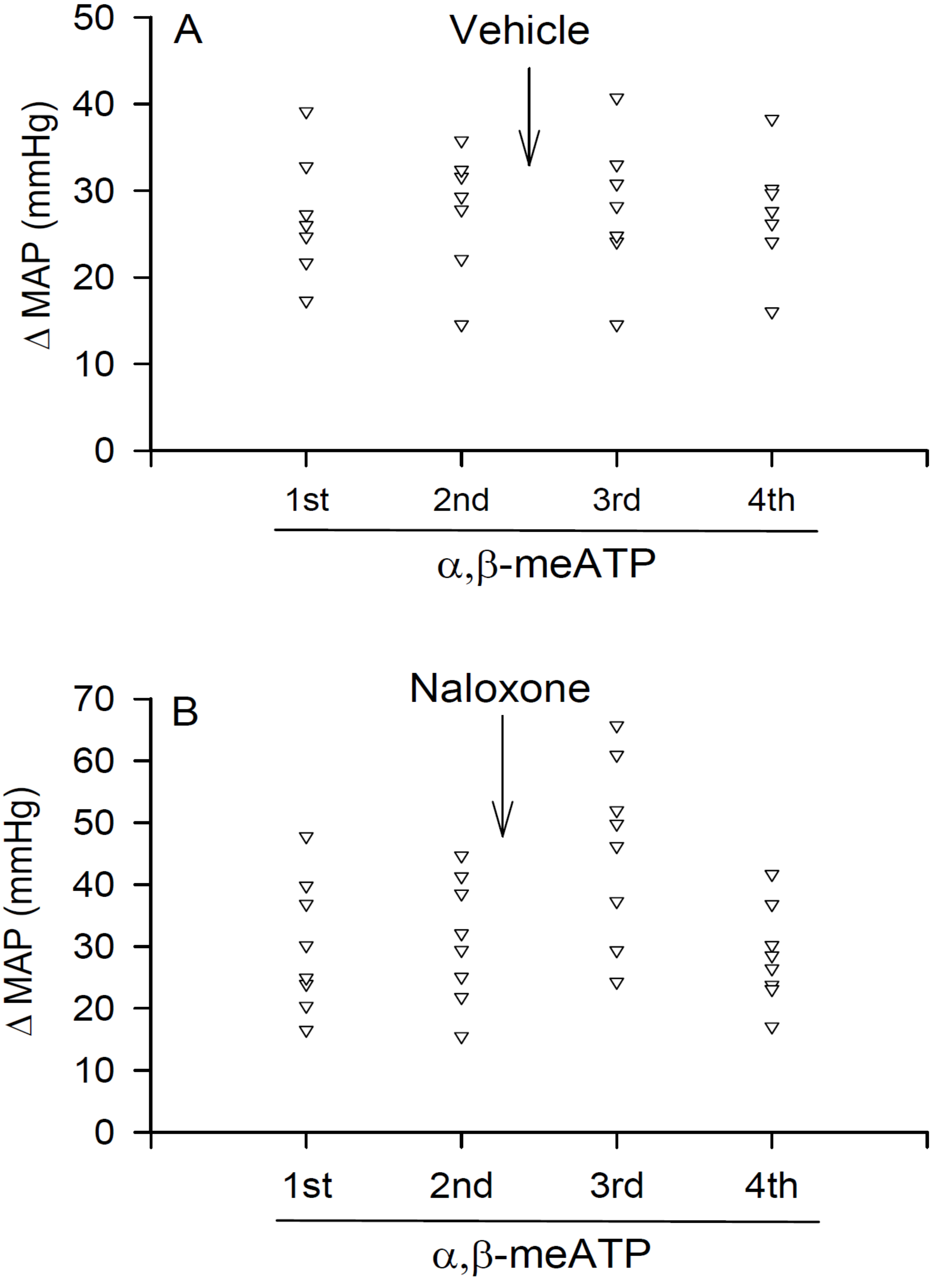

Fig. 5. Individual data point graphs show MAP responses to repeat intrapericardial α,β-meATP (125 nmol) before and after intrapericardial application of vehicle (PBS, n=7, Panel A) and naloxone (n=8, Panel B), a specific opioid receptor antagonist in vagus-intact rats.
